# CsHY5 Regulates Light-Induced Anthocyanin Accumulation in *Camellia sinensis*

**DOI:** 10.3390/ijms26073253

**Published:** 2025-04-01

**Authors:** Jiahao Chen, Yihao Liu, Hongbo Zhao, Jianmei Xu, Peng Zheng, Shaoqun Liu, Binmei Sun

**Affiliations:** College of Horticulture, South China Agricultural University, Guangzhou 510642, China; cjhtea00@stu.scau.edu.cn (J.C.); scauxinyve@163.com (Y.L.); zhao@scau.edu.cn (H.Z.); xu15070368164@163.com (J.X.); zhengp@scau.edu.cn (P.Z.)

**Keywords:** light, *CsHY5*, anthocyanin biosynthesis, shading, *Camellia sinensis*

## Abstract

Tea is one of the world’s major non-alcoholic beverages, popular for its health benefits and flavor. Purple-bud tea is particularly rich in anthocyanins, the concentration of which varies depending on the tea cultivar and cultivation conditions. While the genetic regulation of anthocyanin accumulation is well understood, the impact of environmental factors, such as light, on anthocyanin synthesis is less documented. In this study, we analyzed the anthocyanin content and the expression levels of *anthocyanin biosynthesis genes (ABGs)*, *CsAN1*, and *CsHY5*, under different light intensities and durations. The expression of both *CsAN1* and *ABGs* was significantly induced by light, with an intensity of 8000 lx particularly effective in promoting anthocyanin accumulation. Furthermore, we explored the effect of shading on anthocyanin content, finding that fifty percent shading reduced anthocyanin content by nearly half. Finally, dual-luciferase reporter assays and yeast one-hybrid assays confirmed the direct regulation of *CsHY5* on *CsAN1*. These findings offer insights into the regulatory mechanisms underlying light-induced anthocyanin biosynthesis in tea plants and suggest a potential method for controlling anthocyanin accumulation in tea production.

## 1. Introduction

Tea (*Camellia sinensis* L.) is the most widely consumed non-alcoholic beverage after water in the world and is an economically and culturally significant crop for its health benefits and appealing flavor [1,2]. In recent years, purple-bud tea has emerged as popular among consumers for its extended health benefits, which is attributable to anthocyanin accumulation [3,4]. The anthocyanin content of tea plants depends on both the cultivar (e.g., ‘Zijuan’ [ZJ]) and environmental factors during cultivation [5]. ‘Zijuan’ (*C. sinensis* var. *assamica* cv. Zijuan), a unique tea variety, accumulates anthocyanin levels, reaching 707 ± 28 μg/g dry weight (cyanidin-3-O-β-D-glucoside equivalent) [6].

Anthocyanins are important lyochromes belonging to a class of phenylpropanoid compounds called flavonoids [7]. Anthocyanins provide a plant with color (red, blue, and purple), which attracts insect pollination. As natural antioxidants, anthocyanins have garnered attention not only for their role in plant physiology but also for their potential applications in the food and pharmaceutical industries [8]. For instance, anthocyanin-rich extracts from blueberries and grapes are widely used as nutraceuticals due to their anti-inflammatory and anti-cancer properties [9,10]. These factors have understandably sparked interest in understanding the regulatory systems controlling anthocyanin biosynthesis.

Anthocyanin biosynthesis occurs within the flavonoid branch of the phenylpropanoid pathway, and the key catalyst enzymes have been identified [11,12]. Biosynthesis of anthocyanin is governed at the transcriptional level by the MYB-bHLH-WD40 (MBW) complex [13]. In our previous studies, an R2R3-MYB transcription factor (TF), CsAN1, was reported to regulate the anthocyanin late biosynthetic genes (LBGs) and confer ectopic accumulation of pigment in purple tea [3]. *CsMYBPA1* [14], *CsMYB113* [15], and *CsMYB90* [16] also regulate anthocyanin biosynthesis in *Camellia sinensis*.

Anthocyanin biosynthesis is induced by environmental stimuli, such as temperature, water, salinity, and light [17,18,19]. The accumulation of anthocyanin is significantly affected by light in several plant species, and light causes tender green tea leaves to turn a red-purple color [19,20,21,22,23], but the underlying mechanisms are poorly understood. Light signals are sensed by photoreceptors and activate various signal transduction pathways. *ELONGATED HYPOCOTYL 5* (*HY5*), a bZIP transcription factor, is the central regulator of the light-responsive signal transduction pathway [24]. Photoreceptors are activated when exposed to light, and these activated photoreceptors directly or indirectly modify the stability of primary TFs, such as HY5, and up-regulate its expression, causing anthocyanin accumulation, while HY5 is ubiquitinated and degraded by the COP1/SPA complex under dark conditions [25,26,27]. *HY5* acts as a master regulator to increase anthocyanin accumulation by binding to promoters of anthocyanin biosynthesis genes or the R2R3-MYB transcription factor [28]. Previous studies found that flavonol content is enhanced by ultraviolet B radiation and reduced by shading through the CsbZIP1-CsMYB12 coordinated activator–repressor network [29]. However, the role of CsAN1 and the underlying stimulatory effects of light on anthocyanin synthesis remains unclear. Furthermore, the agronomic practice of shading, commonly used to enhance tea quality, presents a practical avenue to manipulate anthocyanin levels, yet its molecular basis requires deeper investigation.

In this study, we systematically analyzed anthocyanin accumulation patterns and expression profiles of key anthocyanin biosynthesis genes (ABGs), *CsAN1*, and *CsHY5* under varying light intensities and durations. We further evaluated the practical implications of shading, a common agronomic practice, on anthocyanin content. Through biochemical assays, we demonstrated direct regulatory interactions between *CsHY5* and *CsAN1*. Our findings provide mechanistic insights into light-induced anthocyanin biosynthesis in tea plants.

## 2. Results

### 2.1. CsHY5 and ABGs Were Significantly Induced by Light

**Figure 1:**
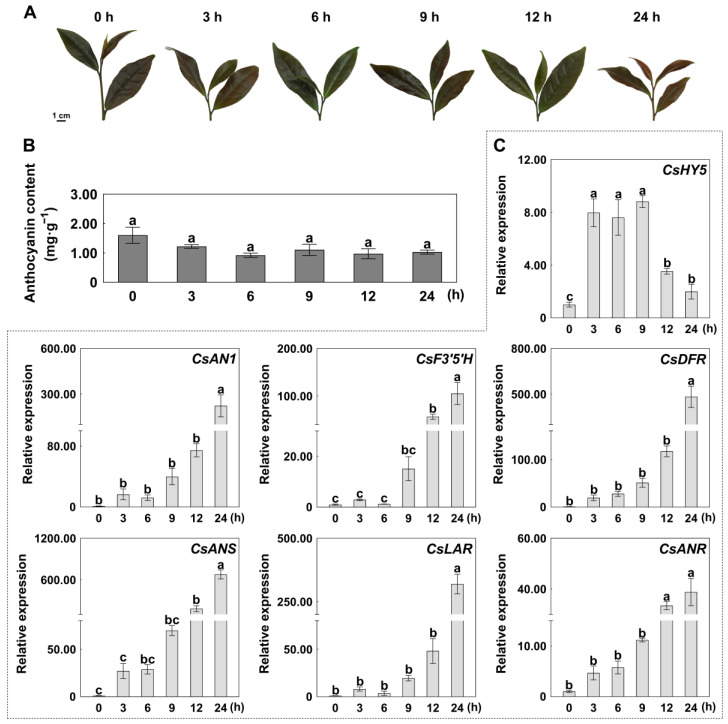
Effect of light exposure duration on anthocyanin biosynthesis in *Camellia sinensis*. (**A**) Tender leaves of ‘ZJ’ tea under different light duration treatments (0, 3, 6, 9, 12, and 24 h). (**B**) Total anthocyanin content under different light duration treatments. (**C**) Expression levels of *CsHY5*, *CsAN1*, and anthocyanin biosynthesis genes (ABGs) under different light duration treatments. Error bars are the SD for three replicate reactions; different letters indicate significant differences determined by the Student’s *t*-test.

To study the effect of light induction on anthocyanin accumulation, tea plants were dark-adapted (72 h) and then illuminated, and samples were collected at 0, 3, 6, 9, 12, and 24 h post-exposure, respectively. Three independent biological replicates for each treatment, with each replicate consisting of three individual plants, were performed to ensure the reliability of our results. The total anthocyanin content and expression levels of related genes, such as *CsHY5*, *CsAN1*, *CsF3′5′H*, *CsDFR*, *CsANS*, *CsANR*, and *CsLAR*, were measured. Short light exposure durations had no significant effect on anthocyanin content (Figure 1A,B). However, the expression levels of both *CsAN1* and ABGs (*CsF3′5′H*, *CsDFR*, *CsANS*, *CsLAR*, and *CsANR*) were significantly induced by light. The highest expression levels were observed at 24 h and then decreased substantially at 9 h (Figure 1C). The expression level of CsHY5 was measured. The expression of *CsHY5* in response to light occurred prior to the expression of *CsAN1* and *ABGs*, suggesting a hierarchical regulatory cascade where CsHY5 activation precedes the transcriptional activation of downstream targets.

### 2.2. Expression Patterns of CsHY5 and ABGs in Response to Light Intensity

**Figure 2:**
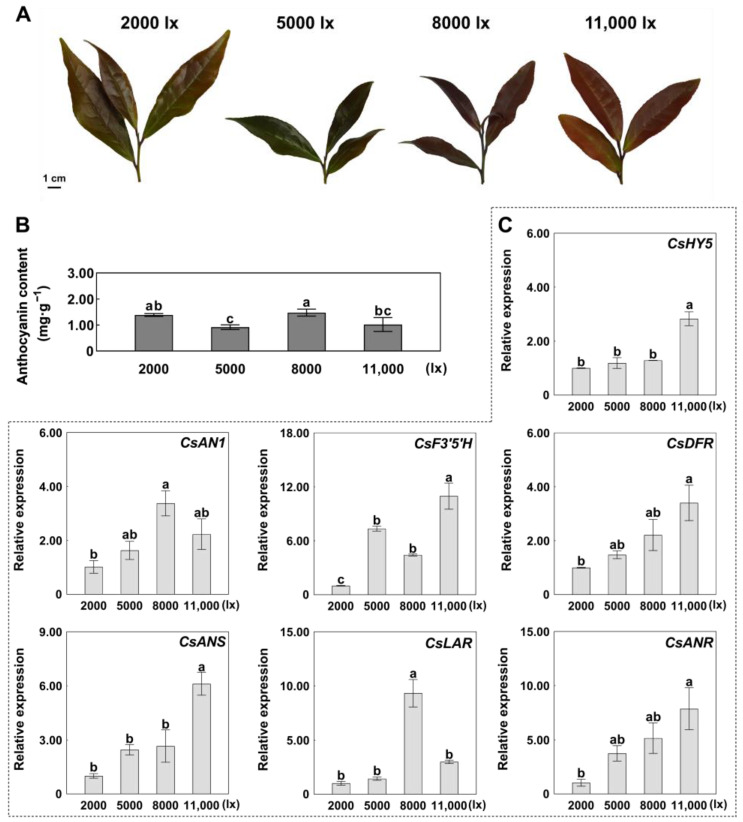
Anthocyanin biosynthesis response to light intensity in *Camellia sinensis*. (**A**) Tender leaves of ‘ZJ’ under different light intensity treatments (2000, 5000, 8000, and 11,000 lx). (**B**) Total anthocyanin content under different light intensity treatments. (**C**) Expression levels of *CsHY5*, *CsAN1*, and anthocyanin biosynthesis genes (ABGs) under different light intensity treatments. Error bars are the SD for three replicate reactions; different letters indicate significant differences determined by the Student’s *t*-test.

Tea plants were exposed to graded light intensities (2000, 5000, 8000, and 11,000 lx) for 72 h in controlled growth chambers. Total anthocyanin contents (mg/g) were 1.39, 0.92, 1.48, and 1.02, respectively, under 2000, 5000, 8000, and 11,000 lx light intensity treatments (Figure 2A,B). Anthocyanin accumulation showed a nonlinear response to light intensity, peaking at 8000 lx. Transcriptional profiling revealed divergent regulatory patterns; the expression levels of *CsHY5* and most ABGs, such as *CsF3′5′H*, *CsDFR*, *CsANS*, and *CsANR*, were highest under the 11,000 lux treatment (Figure 2C). However, *CsAN1* and *CsLAR* had the highest expression level at 8000 lx, consistent with when anthocyanin content was highest (Figure 2B,C). These results implied that the relationship between the expression levels of *CsHY5*, *CsAN1*, and ABGs is complex. For example, the highest expression level of CsHY5 was under the 11,000 lux treatment, and the expression levels of *CsAN1*, *CsLAR*, and *CsANR* did not reach the highest simultaneously (Figure 2C). Discordance between transcript levels (11,000 lx maxima) and metabolite accumulation (8000 lx peak) implies potential feedback regulation.

### 2.3. Shading-Mediated Suppression of Anthocyanin Accumulation

Tea cultivation under shade, also referred to as ‘covering’, is a traditional and effective method in tea agriculture to increase the quality of green tea. Covering makes tea leaves more tender, enhances their green color, increases umami, and reduces bitterness/astringency. The influence of shading intensity on chlorophyll, carotenoid, and metabolite biosynthesis in green tea has been well documented [30]. However, whether shading is an effective practice to control anthocyanin content is unclear.

**Figure 3:**
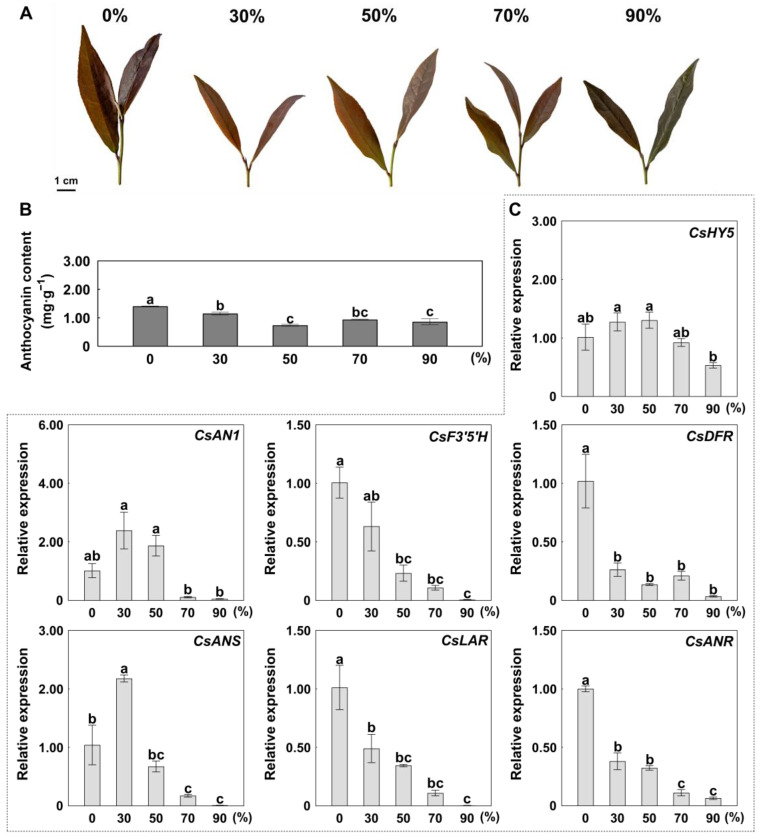
The effect of different shading treatments on anthocyanin content. (**A**) Tender leaves of ‘ZJ’ under different shade treatments (0, 30, 50, 70, and 90% shade). (**B**) Total anthocyanin content under different shade treatments. (**C**) Expression levels of *CsHY5*, *CsAN1*, and anthocyanin biosynthesis genes (ABGs) under different shade treatments. Error bars are the SD for three replicate reactions; different letters indicate significant differences determined by the Student’s *t*-test.

To explore the effect of shading on anthocyanin content, we compared tea plants grown in 30, 50, 70, and 90% shade to those grown under natural light intensity (0% shade). Results showed that shading significantly reduced anthocyanin content (Figure 3A,B). In particular, the total anthocyanin content of the 50% shade treatment was the lowest, with a 47% decrease in total anthocyanin compared to the control (Figure 3B). Meanwhile, the expression levels of ABGs, such as *CsF3′5′H*, *CsDFR*, *CsLAR*, and *CsANR*, were significantly down-regulated compared to the control (Figure 3C). However, the expression levels of *CsHY5* and *CsAN1* did not differ significantly among shade treatments (Figure 3C).

### 2.4. CsHY5 Controls Anthocyanin Biosynthesis by Directly Regulating CsAN1 Expression

**Figure 4:**
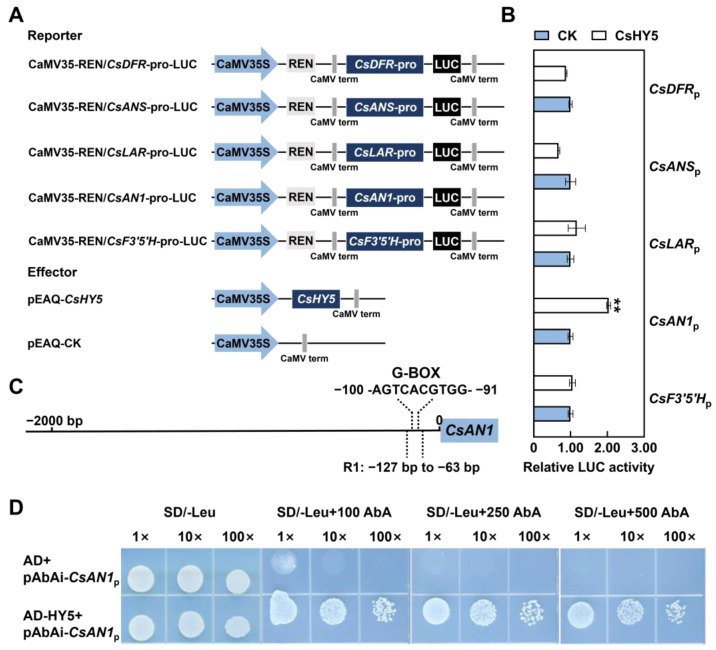
CsHY5 directly regulated *CsAN1* to control anthocyanin content. (**A**) Reporter and effector expression vectors used for dual-luciferase assays. (**B**) Transactivation of CsHY5 on the promoter activity of *CsAN1*, *CsF3′5′H*, *CsDFR*, *CsANS*, and *CsDFR* in the luciferase reporter assay. (**C**) Element analysis of the *CsAN1* promoter. The G-box element included on the *CsAN1* promoter is indicated. (**D**) CsHY5 bound to the promoter of *CsAN1* as determined by yeast one-hybrid assays. Error bars are the SD for three replicate reactions; ** indicate significant differences determined by the Student’s *t*-test.

*CsHY5* cloned from ‘ZJ’ shared 77.55% sequence similarity with *AtHY5* (*Arabidopsis*) and clustered together with *AcHY5* (*Actinidia chinensis*) with 78.61% similarity at the amino acid sequence level (Supplementary Figure S1A). Furthermore, *CsHY5* had a conserved domain and a basic leucine zipper domain (bZIP) (Supplementary Figure S1B). The subcellular location assay showed that the GFP-CsHY5 fusion protein signals were observed in the tobacco epidermal cell nucleus, suggesting that CsHY5 is localized to the nucleus (Supplementary Figure S1C). Moreover, the dual-luciferase reporter assay results showed that CsHY5 markedly activated the promotor of *CsAN1* but had no activation effect on the promotors of ABGs, such as *CsF3′5′H*, *CsDFR*, *CsANS*, and *CsDFR* (Figure 4A,B). The promotor of *CsAN1* had a G-box, which could be recognized specifically by the bZIP transcription factor (Figure 4C). Y1H assays revealed that CsHY5 bound to the promoter of *CsAN1* (Figure 4D), which suggests that *CsAN1* directly regulates anthocyanin content in *Camellia sinensis*.

## 3. Discussion

### 3.1. Light Influenced Anthocyanin Biosynthesis

Anthocyanin biosynthesis is induced by many environmental stimuli; for example, ‘Zijuan’ tea exhibits higher anthocyanin levels under cool conditions [31]. However, light is an important environmental factor affecting anthocyanin biosynthesis [25,26,27]. In apple (*Malus domestica*), the activity of MdMPK6 was increased under light conditions and elevated the phosphorylation level of MdHY5, which enhances the binding of MdHY5 to genes related to anthocyanin synthesis [32]. Under UV-B light conditions, the expression of ZbHY5 in *Zanthoxylum bungeanum* is up-regulated, and it co-regulates with ZbMYB113 to modulate the expression of anthocyanin biosynthesis genes, promoting anthocyanin accumulation in the leaves [33]. In addition, the light and temperature stress-regulated anthocyanin could be modulated by CsMYBL2 homologs in tea plants (*Camellia sinensis*) [20].

After 3 days of dark treatment followed by re-illumination, the expression level of *CsHY5* increased between 3 and 9 h of re-illumination (Figure 1C), consistent with previous studies. *CsHY5* was destabilized in darkness, and bright light exposure induced its expression rapidly in tea plants. In addition, the expression levels of *CsAN1* and ABGs (*CsF3′5′H*, *CsDFR*, *CsANS*, *CsLAR*, and *CsANR*) increased along with re-illumination time (Figure 1C). However, anthocyanin content did not significantly increase within the 24 h we observed (Figure 1A). We speculate that short-term light exposure (within 24 h) is long enough to promote the expression of genes related to anthocyanin biosynthesis but not long enough to observe measurable anthocyanin accumulation. Notably, the expression level of CsHY5 was significantly up-regulated at 3 h (Figure 1C), aligning with its role as an early-response transcription factor, which is consistent with the HY5 dynamics observed in Arabidopsis under blue light exposure [34].

The expression patterns of CsHY5 and ABGs responding to light intensity are complex. For example, the highest expression level of CsHY5 was under the 11,000 lx treatment, while the expression levels of *CsAN1*, *CsLAR*, and *CsANR* reached the highest under the 8000 lx treatment (Figure 2C). The non-linear relationship between light intensity and anthocyanin accumulation may reflect a balance between light-driven activation and photoinhibition. High-intensity light (11,000 lx) could induce oxidative stress, leading to the degradation of anthocyanins or inhibition of biosynthetic enzymes [35]. This hypothesis is supported by studies in grape berries, where excessive UV-B radiation reduces anthocyanin stability despite up-regulating biosynthesis genes [36]. Furthermore, the discordance between CsHY5 expression (maximal at 11,000 lx) and anthocyanin content (peaking at 8000 lx) suggests post-transcriptional regulation or feedback inhibition. For instance, HY5 stability might be compromised under prolonged high-light conditions due to increased COP1/SPA-mediated ubiquitination [27]. Future studies could explore the protein-level dynamics of CsHY5 and its interaction with degradation machinery under varying light intensities.

### 3.2. Shading Could Be Used to Control the Anthocyanin Content of Tea Plants

Higher light intensity in summer months results in tea plants with more purple buds and leaves rich in anthocyanins [37], while shading reduces bitterness/astringency and increases tea quality [38]. Ji et al. [39] found that the levels of catechins were influenced by the initial reduction of light provided by shading, while most amino acids increased as the duration of shading increased. Wang et al. [40] revealed that shade had notable effects on flavonoid content (including catechins, O-glycosylated flavonols, and proanthocyanins), but had no significant effect on anthocyanin accumulation. In this study, we found that 30%, 50%, 70%, and 90% shading could significantly reduce anthocyanin content, and 50% shading has the best effect on decreasing total anthocyanins by 47%, along with down-regulation of ABGs (Figure 3B,C). Our results were consistent with research on other plant species in which shade treatments decreased anthocyanin synthesis [41,42]. Therefore, shading could be an effective and convenient agricultural method to control the anthocyanin content in tea production.

### 3.3. CsHY5 Regulated Anthocyanin Biosynthesis via CsAN1 Expression

HY5 regulates anthocyanin biosynthesis by binding to the G-box in the promoter of transcription factors, thereby promoting the accumulation of anthocyanins [28,43]. In *Camellia sinensis*, CsbZIP1 positively regulated CsMYB12 and specifically activated flavonol biosynthesis [29]. In our previous studies, CsAN1 was found to be a key regulator of anthocyanin biosynthesis to regulate the expression of *CsLDOX2* [3]. However, whether CsHY5 regulates *CsAN1* and thereby causes the anthocyanin accumulation in response to light was not clear. In the dual-luciferase reporter assay of the current study, CsHY5 markedly activated the promotor of *CsAN1* instead of the promotors of ABGs (Figure 4B). The following Y1H assays revealed that CsHY5 could bind to the G-box of *CsAN1* promoters (Figure 4C,D). The above results show that the expression of *CsHY5* was induced by light. CsHY5 directly binds to and activates *CsAN1*, which is a regulator of anthocyanin biosynthesis, thereby directly contributing to anthocyanin accumulation in response to light in *Camellia sinensis*. Our findings establish a direct regulatory link between CsHY5 and CsAN1, but several questions remain. Firstly, whether CsHY5 interacts with other components of the MBW complex (e.g., bHLH or WD40 proteins) to fine-tune anthocyanin biosynthesis warrants investigation. In apple, HY5 cooperates with MYB partners to enhance promoter binding efficiency [36]. Secondly, the lack of CsHY5 activation on *ABG* promoters (Figure 4B) implies that CsAN1 alone may suffice to drive *ABG* expression, as shown in purple sweet potato where IbAN1 directly activates structural genes [44].

## 4. Materials and Methods

### 4.1. Plant Growth Conditions

Three-year-old ‘Zijuan’ (ZJ) tea plants were cultivated in the experimental tea garden of South China Agricultural University (Guangzhou, China). Three independent light experiments were conducted as follows:

① Light duration test: Plants were first dark-adapted for 72 h, then exposed to light in an artificial climate chamber (Shengyuan Instrument Co., Zhengzhou, China) for 0, 3, 6, 9, 12, or 24 h;

② Light intensity test: Plants were maintained under continuous illumination at 2000, 5000, 8000, or 11,000 lux for 72 h;

③ A separate shading experiment was conducted at Longyuan Tea Garden (Huizhou, China) with 0% (control), 30%, 50%, 70%, and 90% shading treatments for 120 h.

Tender leaves (pekoe) from all experiments were flash-frozen in liquid nitrogen and stored at −80 °C until analysis. Three biological replicates were performed using distinct plant sets for each treatment.

Nicotiana benthamiana seedlings were grown in controlled conditions: 24 °C, 50–70% relative humidity, and 16/8 h light/dark cycle. Two-week-old leaves were used for transient expression assays (subcellular localization and dual-luciferase reporter assay).

All data are represented as the mean and standard deviation (SD) of three independent biological replicates, each with three technical replicates. Significant differences were detected using a Student’s *t*-test in GraphPad Prism 9.5.0 software.

### 4.2. Anthocyanin Extraction and Analysis

Tender leaves of ‘ZJ’ were ground to a fine powder and freeze-dried (Labconco FreeZone 2.5, Kansas City, MO, USA). Extraction and quantification of anthocyanin was performed with a total anthocyanins content kit (G0126W, Grace Biotechnology, Suzhou, China) according to the instructions provided. Briefly, 0.02 g freeze-dried powder was extracted in 1 mL extraction solution in a 75 °C water bath for 25 min, followed by centrifugation at 12,000 rpm for 10 min; then, 100 μL of the supernatant was mixed with 300 μL of Solution I and incubated in the dark at room temperature for 1 h. Anthocyanins were quantified by microplate photometry (Multiskan™ SkyHigh, Thermo Fisher, Waltham, MA, USA) at 530 nm and 700 nm. The total anthocyanin content was calculated using the formula below:

Total anthocyanins content (mg/g) = 0.1336 × ΔA ÷ W × D(1)

where ΔA = (A_530_ − A_700_)_sample_ − (A_530_ − A_700_)_control_; W was weight of the sample; and D was dilution ratio.

### 4.3. Quantitative Real-Time Polymerase Chain Reaction (qRT-PCR)

Total RNA was isolated from leaves with the Magen HiPure Plant RNA Mini kit B (R4151, Magen, Guangzhou, China), following the manufacturer’s protocol. Briefly, 100 mg of frozen leaf tissue was homogenized in liquid nitrogen using a sterile mortar and pestle, followed by lysis in 1 mL of RLT buffer containing β-mercaptoethanol (1% *v*/*v*) to inhibit RNase activity. RNA purity and concentration were assessed using a NanoDrop 2000 spectrophotometer (Thermo Fisher Scientific, Waltham, MA, USA), with A260/A280 ratios between 1.8 and 2.0 and A260/A230 > 1.5 indicating high-quality RNA. RNA integrity was further verified by 1% agarose gel electrophoresis, showing clear 28S and 18S ribosomal RNA bands without genomic DNA contamination.

Double-stranded cDNA was prepared using the HiScript III RT 1st Strand cDNA Synthesis kit (R323-01, Vazyme, Nanjing, China), following the manufacturer’s instructions. For each reaction, 1 μg of total RNA was reverse-transcribed in a 20 μL reaction volume containing 4 μL of 5× HiScript III Buffer, 1 μL of HiScript III Enzyme Mix, and 1 μL of Oligo(dT)23 VN primer. The reaction conditions were set as follows: 25 °C for 5 min (primer annealing), 50 °C for 15 min (reverse transcription), and 85 °C for 5 min (enzyme inactivation). cDNA samples were diluted 10-fold with nuclease-free water and stored at −20 °C for subsequent qRT-PCR analysis.

The qRT-PCR was carried out using the qPCR SYBR Green Master Mix (Yeasen, Shanghai, China) and detected with the Bio-Rad CFX384 TouchTM system (Bio-Rad, Hercules, CA, USA). Each 10 μL reaction contained 5 μL of SYBR Green Master Mix, 0.5 μL each of forward and reverse primers (10 μM), 1 μL of diluted cDNA template, and 3 μL of nuclease-free water. Thermal cycling conditions were as follows: initial denaturation at 95 °C for 3 min, followed by 40 cycles of 95 °C for 10 s, 60 °C for 30 s, and 72 °C for 15 s. A melt curve analysis (65–95 °C, increment 0.5 °C/5 s) was performed to confirm primer specificity. All reactions included three technical replicates per biological sample, and negative controls (no-template controls) were included to exclude contamination. Primers were designed using Primer Premier 5.0 (Premier Biosoft International, Palo Alto, CA, USA) and are listed in Supplementary Table S1. The 2^−ΔΔCT^ method was utilized to estimate the relative transcription values for qRT–PCR normalization using actin (TEA019484.1) as the reference gene [45].

### 4.4. Multiple Sequence Alignment and Phylogenetic Analysis

The full-length deduced amino acid sequences of CsHY5 and its homologs were aligned using CLUSTAL OMEGA (version 1.2.4) with default parameters, including the iterative alignment algorithm (HHalign) for improved accuracy in distantly related sequences. Gap opening and extension penalties were set to 10 and 0.2, respectively, to balance alignment sensitivity and specificity. A phylogenetic tree was constructed via the neighbor-joining method with 1000 bootstrap replicates using the MEGAX64 software v4.8.1 package under the Jones–Taylor–Thornton (JTT) substitution model, which accounts for amino acid substitution rates and is widely recommended for plant protein phylogeny. To validate the robustness of the tree topology, we additionally performed a maximum likelihood analysis using RAxML (version 8.2.12) with 1000 bootstrap replicates and the LG+G+F model, which accommodates site-specific rate heterogeneity and frequency variations. Both methods yielded congruent tree topologies, supporting the reliability of the evolutionary relationships inferred.

The bootstrap values, representing the percentage of replicate trees supporting each branch, were annotated on the consensus tree. Branches with bootstrap values ≥ 70% were considered strongly supported. The final tree was visualized and annotated using FigTree (version 1.4.4), with branch lengths scaled to represent genetic distances. To contextualize the evolutionary position of CsHY5, we included HY5 homologs from diverse plant species, such as *VvHY5* (*Vitis vinifera*), XP_010648648.1; AtHY5 (*Arabidopsis thaliana*), NP_001330553.1; AcHY5 (*Actinidia chinensis*), PSR91830.1; TcHY5 (*Theobroma cacao*), XP_007013841.2A; MdHY5 (*Malus domestica*), NP_001280752.1.

### 4.5. Subcellular Localization

The coding sequence (CDS) of CsHY5 was cloned from ‘ZJ’. The open reading frame (ORF) of CsHY5 was cloned into the pEAQ-EGFP vector, pEAQ-EGFP-CsHY5, for subcellular localization studies. The empty vector (pEAQ-EGFP) was used as a control. Plasmids were introduced into *Agrobacterium tumefaciens* strain GV3101 by infiltration of two-week-old *Nicotiana benthamiana* leaves. After 48 h of infiltration, green fluorescence protein (GFP) fluorescence signals were examined using an Axio Imager D2 microscope (Carl Zeiss, Oberkochen, Germany).

### 4.6. Yeast One-Hybrid Assays (Y1H)

The ORFs of CsHY5 were cloned in-frame after the transcriptional activation domain of yeast GAL4 in the pGADT7 vector to generate a prey vector, AD-CsHY5. The promoter fragment of *CsAN1* was cloned into the pAbAi vector as a bait vector, pAbAi-ProCsAN1. Then, the recombination plasmid of pAbAi-ProCsAN1 was transformed into the yeast strain Y1H gold (Coolaber, Beijing, China) and cultured on synthetic defined (SD) medium lacking uracil (SD/−Ura) with 100, 200, 500, 800, and 1000 ng/mL Aureobasidin A (AbA) to select a concentration that inhibited background expression. pAbAi-ProCsAN1+ AD-CsHY5 were co-transformed into the yeast strain Y1H gold and cultured on SD medium lacking leucine (SD/−Leu) with varying AbA concentrations, as described previously. pAbAi-P53+ pGADT7-53 and pAbAi-ProCsAN1+ pGADT7 were used as positive and negative controls, respectively. The co-transformed yeast was grown at 29 °C for 48 h.

### 4.7. Dual-Luciferase Reporter Assay

The 35S:CsHY5 construct was cloned into the pEAQ-62SK vector as an effector, and the promoter fragments of CsAN1, CsF3′5′H, CsDFR, CsANS, and CsLAR were cloned into pGreenII 0800-LUC vectors as reporters. The effector and reporters were transformed into *A. tumefaciens GV3101* (p-soup) (Wego, Shanghai, China). The effector and reporter constructs were co-injected into three-week-old *N. benthamiana* leaves and cultured for 48 h in the shade. The Dual-Luciferase^®^ Reporter (DLR™) Assay System (Promega, Madison, WI, USA) was used to detect LUC/REN activity using Cytation5 Enzyme Labeler (Biotek, Winooski, Germany), following the manufacturer’s instructions.

## 5. Conclusions

The expression of both *CsAN1* and *ABGs* was significantly induced by an intensity of 8000 lx, which was particularly effective in promoting anthocyanin accumulation. Fifty percent shading could significantly reduce anthocyanin content by nearly half. Dual-luciferase reporter assays and yeast one-hybrid assays confirmed the direct regulation of *CsHY5* on *CsAN1*. These insights not only advance our understanding of light-regulated anthocyanin biosynthesis but also provide actionable strategies for optimizing tea cultivation. For instance, adjusting shading levels during specific growth stages could control the anthocyanin content in tea production.

## Data Availability

All the data supporting the findings of this study are available in the article and Supplementary Materials.

## Supplementary Materials

The following supporting information can be downloaded at https://www.mdpi.com/article/10.3390/ijms26073253/s1.
