# Supplementary material for: CsHY5 Regulates Light-Induced Anthocyanin Accumulation in Camellia sinensis
Source: Int J Mol Sci. 2025 Apr 1;26(7):3253. doi: 10.3390/ijms26073253 (PMC11989487; doi:10.3390/ijms26073253)
Supplement: Supplementary file 1 [file ijms-26-03253-s001.zip › ijms-3505334-supplementary.pdf]

Supplemental Table S1. The primer list.

Gene name	Primers sequence (5'-3')
qPCR-CsActin-F	GCCATATTTGATTGGAATGG
qPCR-CsActin-R	GGTGCCACAACCTTGATCTT
qPCR-CsHY5-F	GATGAGATCAGGAGAGTGCCG
qPCR-CsHY5-R	CTTGTTTTCCTTGTCAGCCGG
qPCR-CsAN1-F	CTAGGAAACAGGCAAGTGCG
qPCR-CsAN1-R	GAGAAGGTTCGAGGTCGAGG
qPCR-CsF3'5'H-F	AAAGTAATTGGAAGAAACCGCC
qPCR-CsF3'5'H-R	TAGGAATACTGAGGGGAACTGA
qPCR-CsDFR-F	ATGAAAGACTCTGTTGCTTCTG
qPCR-CsDFR-R	TGCTTCACCTTCTTTAAATTCG
qPCR-CsLAR-F	GTGTTGGAATCTGTGTCCGCAG
qPCR-CsLAR-R	CTCCATGAATGCTTGATCCTTG
qPCR-CsANR-F	AACCAGCAATTCAAGGAGTAGT
qPCR-CsANR-R	TCCCATTGAGCTTATTGATCGA
qPCR-CsANS-F	CTCCATCGTGGACTCGTTAATA
qPCR-CsANS-R	AGAATGATCTTCTCCTTGGGTG
pEAQ-EGFP-CsHY5-F	ctgcccaaattcgcgaccggtATGCAAGAACAAGCAACGAGTTC
pEAQ-EGFP-CsHY5-R	gcccttgctcaccataccggtCTTCCTACCCTCCTGCATTCC

pEAQ-CsHY5-F	ctgcccaaattcgcgaccggtATGCAAGAACAAG CAACGAGTTC
pEAQ-CsHY5-R	gtgatggtgatgcataccggtCTACTTCCTACCCT CCTGCATTCC
pGreen-0800-LUC-CsF3'5'Hpro-F	ttcctgcagcccgggggatccCTCGAGTGGTAAA TGTTGCGC
pGreen-0800-LUC-CsF3'5'Hpro-R	tgtttttggcgtcttccatggTGGGGATAGTTTGAA CTAGGGC
pGreen-0800-LUC-CsDFRpro-F	ttcctgcagcccgggggatccGTAATCGTACTTGT AGATCAAGAAGAGCA
pGreen-0800-LUC-CsDFRpro-R	tgtttttggcgtcttccatggGATGGGATTAAAATG TGAAAAAATTG
pGreen-0800-LUC-CsANSpro-F	ttcctgcagcccgggggatccCCATTTTCCAGGG TTATTGTCTCA
pGreen-0800-LUC-CsANSpro-R	tgtttttggcgtcttccatggAGGGTAAACTGTTAC GCCAGCT
pGreen-0800-LUC-CsLARpro-F	ctatagggcgaattgggtaccGCGTGTAACTAGT GTATTATGGGCA
pGreen-0800-LUC-CsLARpro-R	tgtttttggcgtcttccatggTCTTTTCGTTACGTA CC TTCCTCTAG
pGreen-0800-LUC-CsAN1pro-F	ttcctgcagcccgggggatccACCCTCATCTGATG AACGTTCTAAG

pGreen-0800-LUC-CsAN1pro-R	tgtttttggcgtcttccatggTCCTCAGTCCATGCACCTTTTC
pAbAi-ProCsAN1-F	aaatgatgaattgaaaagcttGTCATTTAAGGAAGCTGTACCTGAAC
pAbAi-ProCsAN1-R	gtcgacagatccccgggtaccGTCTGAATAAGTAGACTTCCTTGCGC
pGADT7-CsHY5-F	gccatggaggccagtgaattcATGCAAGAACAAGCAACGAGTTC
pGADT7-CsHY5-R	cagctcgagctcgatggatccCTACTTCCTACCCTCCTGCATTCC

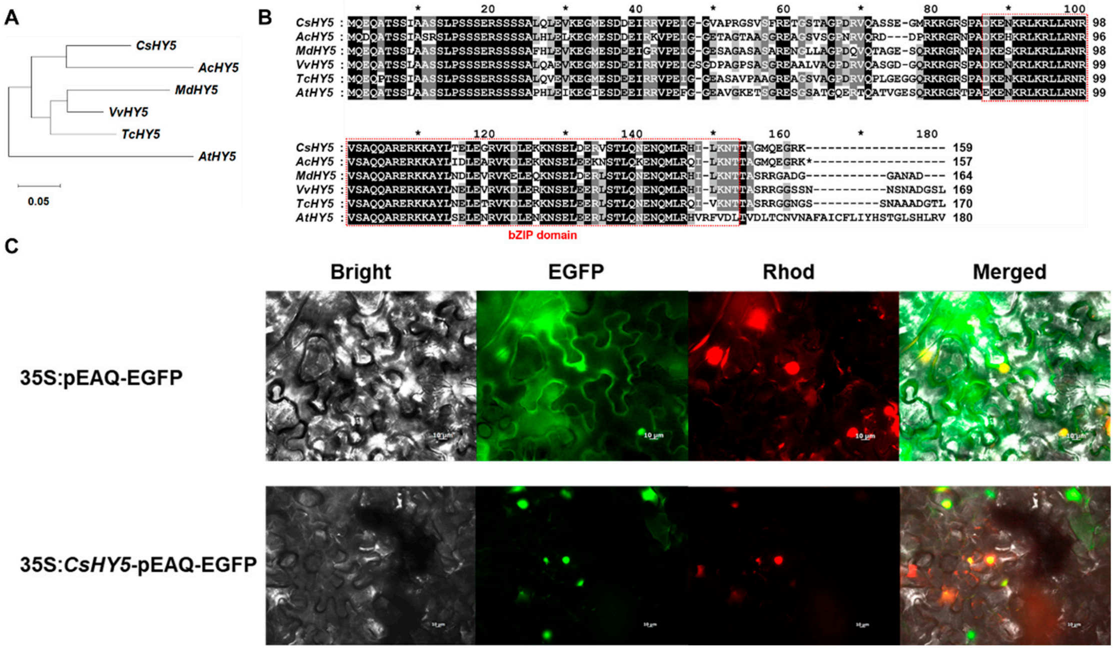

Supplemental Figure S1. Phylogenetic analysis and subcellular location assay of CsHY5. (A) Phylogenetic tree showing the relationship between CsHY5 and its closest homologues. (B) Alignment of the deduced amino acid sequence of CsHY5 using ESPript 3.0. The conserved domain of bZIP transcription factor was indicated by red box. (C) Subcellular location of CsHY5. Accession IDs (GenBank): CsHY5 (Camellia sinensis); VvHY5 (Vitis vinifera), XP_010648648.1; AtHY5 (Arabidopsis thaliana) NP_001330553.1; AcHY5 (Actinidia chinensis) PSR91830.1; TcHY5 (Theobroma cacao) XP_007013841.2A; MdHY5 (Malus domestica) NP_001280752.1.
